# The effect of exercise training on skin blood flow: a systematic review and meta-analysis

**DOI:** 10.1186/1757-1146-8-S2-O23

**Published:** 2015-09-22

**Authors:** Sean M Lanting, Nathan A Johnson, Michael K Baker, Ian D Caterson, Vivienne H Chuter

**Affiliations:** 1 School of Health Sciences, University of Newcastle, Australia; 2 Discipline of Exercise and Sport Science, University of Sydney, Australia; 3 Charles Perkins Centre, University of Sydney, Australia; 4 School of Exercise Science, Australian Catholic University, Australia; 5 Priority Research Centre for Physical Activity and Nutrition, University of Newcastle, UK

**Keywords:** Skin blood flow, microvascular disease, exercise

## Background

Microvascular disease results in reduced skin blood flow (SkBF) and an increased risk of poor healing, ulceration and amputation, particularly in the lower extremity. Regular exercise is known to produce significant cardiovascular benefits and improved functional outcomes in people with chronic disease. However, it is unknown if these benefits also translate into improvements in SkBF. The purpose of this study was to evaluate the efficacy of exercise training on altering SkBF in adults by systematic review and meta-analysis.

## Methods

Relevant databases were searched to July 2014 for controlled trials evaluating the effect of exercise training interventions versus a non-exercise control on SkBF in adults.

## Results

Eight studies met the inclusion criteria for this review. Individual studies employing an exercise intervention tended to have small sample sizes, with six of the eight studies showing a benefit of exercise but only three reaching statistical significance. Subsequent meta-analysis demonstrated aerobic exercise had a statistically significant effect on improving SkBF (ES = 0.49, 95% CI: 0.12 to 0.87, p = 0.010).

## Conclusions

To date, individual studies employing an exercise intervention have lacked sufficient power to detect clinically relevant benefits to SkBF, partially due to limited sample size. In primarily healthy previously sedentary adult cohorts, pooled analysis revealed a clear benefit of regular aerobic exercise on improving SkBF. Regular aerobic exercise provides a cost-effective option for improving SkBF in adults and may be of benefit to those with cardiovascular disease and metabolic disorders such as diabetes.

